# Detection of SARS-CoV-2 in Wastewater at Residential College, Maine, USA, August–November 2020

**DOI:** 10.3201/eid2712.211199

**Published:** 2021-12

**Authors:** Yolanda M. Brooks, Bailey Gryskwicz, Shawn Sheehan, Sheri Piers, Parag Mahale, Susan McNeil, Jenna Chase, Doreen Webber, David Borys, Michael Hilton, Dion Robinson, Stephen Sears, Emer Smith, Emily K. Lesher, Robert Wilson, Matthew Goodwin, Michael Pardales

**Affiliations:** Saint Joseph’s College of Maine, Standish, Maine, USA (Y.M. Brooks, B. Gryskwicz, S. Sheehan, S. Piers, S. McNeil, J. Chase, D. Webber, D. Borys, M. Hilton, D. Robinson, E.K. Lesher, R. Wilson, M. Goodwin, M. Pardales);; Centers for Disease Control and Prevention, Atlanta, Georgia, USA (P. Mahale);; Maine Center for Disease Control and Prevention, Augusta, Maine, USA (P. Mahale, S. Sears, E. Smith);; University of Southern Maine, Portland, Maine, USA (E. Smith)

**Keywords:** COVID-19, SARS-CoV-2, respiratory infections, severe acute respiratory syndrome coronavirus 2, 2019 novel coronavirus disease, coronavirus disease, zoonoses, viruses, coronaviruses, wastewater, wastewater surveillance, nucleic acid detection, Maine, United States, students, colleges, residential colleges, surveillance

## Abstract

We used wastewater surveillance to identify 2 coronavirus disease outbreaks at a college in Maine, USA. Cumulative increases of >1 log_10_ severe acute respiratory syndrome coronavirus 2 RNA in consecutive 24-hour composite samples preceded the outbreaks. For 76% of cases, RNA was identified in grab samples from residence halls <7 days before case discovery.

Wastewater surveillance can indicate the presence and temporal trends of coronavirus disease (COVID-19) cases in a sewershed (*1*,*2*). Large universities have used wastewater surveillance to identify residence halls at high risk for transmission of severe acute respiratory syndrome coronavirus 2 (SARS-CoV-2), the causative agent of COVID-19 (*3*,*4*). We demonstrate that wastewater surveillance using grab samples collected from residential halls and 24-hour composite samples from lift stations can detect COVID-19 outbreaks at a small residential college.

## The Study

During August 21–November 20, 2020 (days 0–92), we collected weekly grab samples of flowing untreated wastewater from 6 residence halls at a residential college in Maine, USA. The residence halls served 605 students; hall A housed 64 students, hall B housed 127 students, hall C housed 80 students, hall D housed 109 students, hall E housed 87 students, and hall F housed 138 students. During days 13–92 we also collected 24-hour composite samples approximately twice a week from 2 lift stations (i.e., L1 and L3) where wastewater from various buildings on campus was consolidated in holding tanks and pumped to septic tanks; these composite samples represented the total population in the residence halls. L1 contained effluent from halls E–F and L3 contained effluent from halls A–D. Both lift stations also contained effluent from other campus buildings. The wastewater was collected and stored at 4°C for <72 hours before we assayed 105-mL samples using the Water SARS-CoV-2 RT-PCR test (IDEXX Laboratories, Inc., https://www.idexx.com) according to the manufacturer’s instructions. The 2019-nCoV_N_Positive Control plasmid (Integrated DNA Technologies, Inc., https://www.idtdna.com) had a limit of quantification of 2 (average cycle threshold [C_t_] 39.42) copies per reaction, and the purified 2019-nCoV_N_Positive Control plasmid cloned into *Escherichia coli* had a limit of quantification of 20 (average C_t_ 35.95) copies per reaction. The theoretical limit of detection in all samples was 1 copy per reaction. We included a negative extraction control, no-template control, and a step from the standard curve (2 × 10^3^ or 2 × 10^4^ copies/reaction) in each run. We calculated SARS-CoV-2 RNA concentrations as copies per day per person for 24-hour composite samples and copies per liter per person for grab samples. Nondetectable samples were reported at one half the theoretical limit of detection (*5*).

Each week, ≈100 students were randomly selected for individual surveillance testing by reverse transcription PCR. We instituted expanded surveillance testing for students living in residence halls with detectable RNA in grab samples or served by lift stations that had increased RNA concentrations.

Affected students were isolated or quarantined according to guidelines from the Centers for Disease Control and Prevention (*6*,*7*). COVID-19 outbreak investigations by the Maine Center for Disease Control and Prevention (Augusta, Maine, USA) end 28 days (2 infectious periods) after the specimen collection date of the last identified case. All residential students were tested for SARS-CoV-2 upon outbreak identification, beginning on day 25 for the first outbreak and day 81 for the second outbreak. Widespread testing among students continued weekly until all students had negative test results for 2 successive rounds during the outbreak period.

On day 18, we detected SARS-CoV-2 RNA from the wastewater discharge of hall F and both lift stations, preceding an outbreak on days 21–58 (Figure, panels A, B). By day 22, RNA concentrations in both lift stations increased by >1 log_10_; subsequently, concentrations decreased by >1 log_10_ by day 33 for L3 and day 40 for L1 (Figure, panel B). On day 21, expanded individual surveillance testing of half of the students living in hall F identified 2 COVID-19 cases (Figure, panels C, D). Widespread surveillance testing beginning on day 22 identified 6 additional cases in hall F, 1 case in hall D, and 1 case in hall E (Figure, panel C). In response, the college implemented remote learning during days 22–36.

**Figure:**
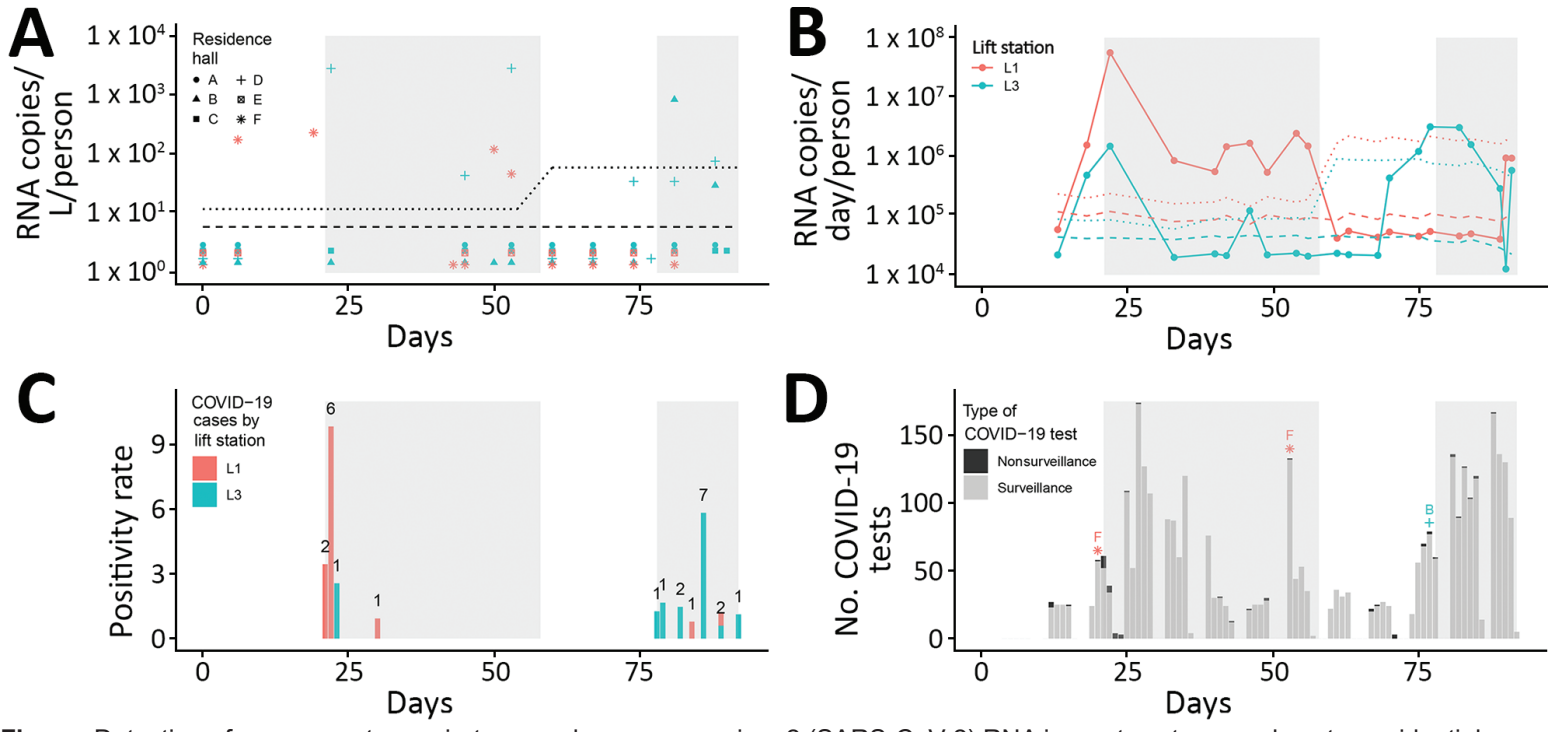
Detection of severe acute respiratory syndrome coronavirus 2 (SARS-CoV-2) RNA in wastewater samples at a residential college, Maine, USA, August–November 2020. A) Grab samples from 6 residence halls (i.e., A–F). B) 24-hour composite samples from 2 lift stations: L1 for halls E–F and L3 for halls A–D. Dashed lines indicate theoretical limit of detection; dotted lines indicate limit of quantification. These limits were dependent on flow rate (B only) and population served for each sample. Data points below dashed lines indicate undetected concentrations of SARS-CoV-2 RNA and are recorded at one-half the limit of quantification (*5*). Data points between dashed and dotted lines indicates detectable but nonquantifiable concentrations of SARS-CoV-2 RNA and are recorded at one half the limit of quantification. C) Daily positivity rate of COVID-19 tests. The number above the bar indicates the number of positive cases. Positive results were typically received within 24 hours after administration of the diagnostic test. D) Total COVID-19 diagnostic tests, including surveillance and nonsurveillance tests. Letter and symbol indicate resident hall of affected student. Shaded areas indicate the days during which the college declared an active outbreak. COVID-19, coronavirus disease.

Students returned to their residence halls from quarantine or isolation on days 21–45; we observed an increase of SARS-CoV-2 RNA in L3 on days 40–46 (Figure, panel B). We detected RNA in grab samples from hall D on days 45 and 53 and hall F on days 50 and 53 **(**Figure, panel A); however, concentrations were undetectable in L3 on days 49–70 (Figure, panel B). On day 53, individual surveillance testing of all students in hall F did not identify any COVID-19 cases (Figure, panels C, D).

From days 70 to 82, SARS-CoV-2 RNA concentrations in L3 increased by >1 log_10_. A second outbreak occurred during days 78–92. Expanded surveillance testing of all students in hall B identified 3 COVID-19 cases, prompting an outbreak investigation. During days 83–89, widespread testing of all students detected 7 additional COVID-19 cases in hall B, 1 case in hall C, and 1 case in hall E (Figure, panel C). The first COVID-19 case in hall E was discovered on day 84, when SARS-CoV-2 RNA was undetectable in L1 (Figure, panel B). The discovery of 7 cases in hall B on day 86 indicated that transmission might be widespread. During days 88–92, the college instituted an exit strategy comprising remote learning and widespread surveillance testing of all students 48 hours before departing campus. On day 91, we detected SARS-CoV-2 RNA in L1 and identified 3 COVID-19 cases (1 in hall A, 1 in hall B, and 1 in hall E; Figure, panels B, C). We detected RNA in grab samples from hall D 4 days before the outbreak as well as during the outbreak and staged dismissal but did not identify any cases in hall D.

## Conclusions

We found that a >1 log_10_ increase of SARS-CoV-2 RNA concentrations in composite samples from a lift station preceded 2 COVID-19 outbreaks at a college campus. RNA concentrations did not substantially increase during the outbreak even as more cases were discovered (Figure, panels B, C). Thus, wastewater surveillance is best used to discover the onset of outbreaks at a small college campus.

We hypothesized that detectable concentrations of SARS-CoV-2 RNA in the wastewater discharge in L1 on days 40–56 (Figure, panel B) were caused by residual shedding among recovering students who were no longer infectious, as described in clinical data (*8*,*9*). In addition, a study in Utah, USA, documented evidence of residual shedding in municipal wastewater systems after a decrease in reported cases (*2*). In L1, SARS-CoV-2 RNA was undetectable 26 days in the wastewater system after the last known case was identified. The decrease of RNA to undetectable levels was consistent with the previously reported median fecal shedding time of 25 days among hospitalized COVID-19 patients (*9*).

In total, 9 of 10 confirmed patients during the first outbreak and 10 of 15 patients during the second outbreak lived in residence halls where RNA was present in grab samples collected <7 days before diagnosis. Similarly, a study at a large college campus found the presence of RNA in grab samples from residence halls had 79.9% positive predictive value of COVID-19 cases within 4 days of collection (*10*). The RNA in 4 grab samples from halls D and F during days 45–53 could be from visitors recovering or actively infectious with SARS-CoV-2; this ambiguity is a limitation of wastewater surveillance. We did not evaluate the magnitude of the RNA concentrations in the grab samples because those samples represent RNA loading in the wastewater flow only at the time of sampling.

In conclusion, wastewater surveillance can indicate changes in SARS-CoV-2 transmission in the student population at a small residential college. Wastewater surveillance can quickly identify outbreaks, localize the detection of COVID-19 cases, and inform the management of resources for clinical surveillance testing.
